# Understanding the Variation within a Dietary Guideline Index Score to Identify the Priority Food Group Targets for Improving Diet Quality across Population Subgroups

**DOI:** 10.3390/ijerph18020378

**Published:** 2021-01-06

**Authors:** Gilly A. Hendrie, Greg Lyle, Chelsea E. Mauch, Joyce Haddad, Rebecca K. Golley

**Affiliations:** 1 Nutrition and Health Program, Health & Biosecurity, CSIRO, Adelaide, SA 5000, Australia; gilly.hendrie@csiro.au (G.A.H.); joyce.haddad@flinders.edu.au (J.H.); 2 School of Public Health, Curtin University, Bentley Campus, Perth, WA 6102, Australia; greg.lyle@curtin.edu.au; 3 Caring Futures Institute, College of Nursing and Health Sciences, Flinders University, Adelaide, SA 5042, Australia; rebecca.golley@flinders.edu.au

**Keywords:** diet quality, dietary index, dietary patterns, interventions, population health, Australia

## Abstract

Globally, population dietary intakes fall below the guideline recommendations and large-scale interventions have had modest success in improving diet quality. To inform the development of more targeted approaches, this study analysed the variations in self-reported data from an online survey of Australian adults collected between 2015 and 2020, to identify common combinations of low scoring components within a dietary guideline index. A low score was defined as meeting less than half the guideline recommendations (a score <50 out of 100). Among 230,575 adults, a single component analysis showed that 79.5% had a low score for discretionary choices, 72.2% for healthy fats and 70.8% for dairy. The combinations approach showed 83.0% of individuals had two to five low scoring components, with men, younger adults aged 18–30 years and individuals with obesity (BMI ≥ 30) more likely to have five or more. The most common dietary pattern combination included low scores for discretionary choices, dairy and healthy fats. There was a considerable but systematic variation in the low scoring components within the dietary patterns, suggesting that interventions with the flexibility to address particular combinations of key food groups across subgroups could be an effective and resource efficient way to improve diet quality in the population.

## 1. Introduction

Population dietary intakes, globally, are failing to meet the dietary guideline recommendations [1,2,3] and are contributing to poor nutrition, high rates of obesity and non-communicable diseases, such as cardiovascular disease, some forms of cancer, and diabetes [4]. National dietary guidelines are designed to improve the health and wellbeing of populations by promoting a pattern of eating to optimise health and reduce the risk of dietary deficiency and chronic disease [5]. Food-based dietary guidelines are generally consistent in their promotion of foods such as vegetables, fruits, wholegrains, and varying amounts of legumes/pulses, eggs, fish, lean meats, and dairy [5,6,7,8]. Guidelines also suggest limiting intakes of foods and beverages higher in saturated fat, salt, added sugars or alcohol, referred to as ‘discretionary choices’ in Australia [5]. However, data from the most recent 2011–2013 Australian National Nutrition and Physical Activity Survey showed that 35% of adults’ daily energy intake was from discretionary choices [9]. This is problematic because discretionary choices have a poor nutrient profile, and can displace healthy foods in the diet [5]. The same survey found that only one in 20 Australian adults consumed adequate amounts of fruit and vegetables, which according to the Australian Dietary Guidelines is 2 serves of fruit per day and 5–6 serves of vegetables per day, depending on gender and age group [10]. Population dietary intake data from the United States, United Kingdom and other countries, show similar patterns of consumption [2,11,12]. Globally, there is a need to develop large-scale interventions that are effective in increasing the compliance of eating habits with population recommendations, delivering health, social and economic benefits [13].

Public health campaigns and large-scale interventions are commonly used to bring awareness to dietary guideline advice and to encourage behaviour change for improving population dietary intake and health [3]. A two part mass media campaign in Australia targeted obesity prevention, with the first phase, Measure-Up, increasing awareness around being overweight and increased risk of chronic disease [14]. Phase two, Swap It Don’t Stop It, focused on making small changes to lifestyle behaviours such as swapping unhealthy options for healthy food and beverage options, and reducing portion size [15]. Prompted recall of these campaigns was moderate, but only one in six people reported a change in behaviour [15]. Other campaigns have largely focused on fruit and vegetable intake. The ‘Go for 2 & 5′ campaign in Australia [16] and 5-a-day style campaign in countries such as the United Kingdom [17], New Zealand [18] and parts of Europe [19] are examples of public health campaigns promoting fruit and vegetable consumption, in line with dietary guidelines. Campaigns such as these have had modest success in improving the fruit and vegetable intake of populations [3,16]. Interventions delivered over the internet or via mobile device have also traditionally focused on increasing fruit and vegetable intake and demonstrated similar improvements in intake [20,21,22]. Fruit and vegetables warrant being the focus of campaigns and interventions. They are a discrete and easily recognisable group of foods to target, and although there are clear health benefits from higher consumption [23], population intakes are persistently low [2,10].

Achieving healthier diets within the population involves more than simply promoting fruits and vegetables. Other healthy food groups and discretionary choices might also be worthy targets for intervention to improve diet quality. Understanding current population intake patterns can help to determine the appropriate dietary targets and population groups most in need of support. Taking a more targeted approach has been shown to be effective. For example, campaigns that focused on specific foods and were targeted toward subgroups of the population based on personal characteristics such as age, culture and markers of socioeconomic status were shown to be more effective in changing consumption patterns, than generic campaigns [3,13]. Targeting interventions in this way requires data driven insights to better understand dietary patterns and the variation within dietary patterns to identify segments of the population with common areas of poor eating habits. These insights can then inform the development of more targeted large-scale campaigns for improving compliance with national dietary guidelines.

Traditionally, national nutrition survey data are used to identify specific food groups where consumption needs to increase or decrease at the population level. In Australia, these surveys are conducted infrequently, about every 10 to 15 years, with the last survey of 9300 adults occurring between 2011 and 2013 [9]. To compliment these data, other surveys can assess dietary intake using short questions or food frequency questionnaires, which are suited to online self-report, allowing for more frequent and widespread data collection [24]. Food-based questionnaires conducted online, lend themselves to comparing intake to food-based dietary guidelines to assess diet quality across large numbers of individuals. Diet quality indices or dietary guideline indices can be applied to these data to synthesise characteristics of dietary patterns into a single score and can be useful in understanding the degree to which individuals or populations’ eating habits comply with a set of dietary guidelines [25]. There are usually many scoring components used to derive an overall dietary guideline index score. Variation in how these components are scored means that there are many ways to achieve a higher total score (i.e., greater compliance with guidelines), depending on which specific recommendations are being met. Examining these component scores in addition to the total score was recommended, to gain a better understanding of diet quality and dietary patterns [26]. Assessing components of diet quality within an index allows for the identification of single, as well as combinations of guideline recommendations not being met amongst individuals within a population. This more nuanced understanding of individual dietary patterns can facilitate the development of more targeted, and possibly more effective and efficient interventions that prioritise key dietary targets across segments of the population, to improve diet quality [25,26,27].

Therefore, the objective of this exploratory study was to identify and describe the number of low scoring components within dietary patterns, among a large sample of Australian adults, and to describe how the number and most common combinations of low scoring components within a dietary guideline index score varied by gender, age group, weight status and diet quality.

## 2. Materials and Methods

The CSIRO (Commonwealth Scientific and Industrial Research Organisation) Healthy Diet Score is a freely available online survey (CSIRO, Australia and Digital Wellness, Sydney, Australia) assessing individuals’ usual food intake across 38-items [25]. The CSIRO Healthy Diet Score survey allows for servings of food groups to be estimated and compliance with dietary guidelines (termed in this paper as “diet score”) to be calculated, using a scoring algorithm [28]. The survey and diet quality index algorithm were shown to provide a valid estimate of overall diet quality in adults [28].

The CSIRO Healthy Diet Score survey asks individuals to report their intake of food groups, consistent with the Australian Dietary Guidelines [5]. Questions asked about intake of fruit (2 items), vegetables (4 items), grain foods (4 items), meat and alternatives (5 items), dairy and substitutes (4 items), healthy fats (2 items), water (1 item) and discretionary choices (11 items). Discretionary choices include foods and beverages high in added sugars, saturated fat, salt and alcohol, such as cholate and confectionary, cakes and biscuits, savoury pies and pastries, takeaway foods, sugar sweetened beverages and alcohol. Individuals reported the frequency of consumption (daily, weekly, monthly, never) and the amount in servings consumed in the timeframe selected. The survey also asks about the variety of fruit, vegetables, grains, meat and dairy consumed (5 items) [25]. The survey also asks questions on gender, year of birth, and self-reported height and weight (for the calculation of Body Mass Index and categorisation of weight status using the World Health Organization cut-offs) [29]. Year of birth was used to calculate age at time of completing the survey, and age groups were created based on those used in the Nutrient Reference Values for Australia and New Zealand [30].

The scoring algorithm of the CSIRO Healthy Diet Score survey assesses the quantity, quality and variety of foods consumed across the nine scoring components [25,31]. The quantity components include discretionary choices, fruit, vegetables, and meat and alternatives, with the amount consumed being compared against age and gender-specific targets. The grains and dairy components assess the quantity and quality of foods consumed; and the healthy fats, beverages and variety components assess only quality. Scores from each component are summed and a total diet score is estimated and presented as a number between zero and 100, where a higher score reflects greater overall compliance with the Australian Dietary Guidelines [5].

The CSIRO Healthy Diet Score survey is a live website, with data collection occurring continuously. The survey began collecting data on the 21 May 2015, and a series of media releases over the following years promoted website visitation. The media releases resulted in exposure on various mediums, including Australian national television channels, printed and online media, and national and local radio stations. This paper describes data collected from the launch date through to the 1 March 2020, with 298,454 surveys commenced during that time. Duplicate survey entries were excluded, with individuals’ first attempt being included for analysis. This left 282,717 unique users, of which 232,287 were complete surveys (*n* = 49,538 or 17.5% of the unique surveys were partially completed and excluded).

As described previously [25], a standard data cleaning protocol was used whereby those with a Body Mass Index less than 13 kg/m^2^ or greater than 97 kg/m^2^, weight less than 13 kg or greater than 250 kg, height less than 1 m or greater than 3 m, or age less than 18 years or greater than 100 years were considered outliers, and thus excluded from analyses (*n* = 1712). Data from a total of 230,575 individuals were included in this analysis. The nature of recruitment resulted in a sample that was under-representative of males and older adults relative to the broader Australian population, so data were weighted by sex and age group to reflect the population characteristics collected as part of the 2016 Australian Census [32].

All participants provided informed consent for their data to be used for research purposes, and this research was approved by the CSIRO Health and Medical Human Research Ethics Committee Low Risk Review Panel (LR29/2016).

### Identifying the Diet Guideline Index Components Most in Need of Intervention

Individuals’ scores on each of the nine scoring components of the CSIRO Healthy Diet Score survey were categorised as either low or high, based on a cut-off of 50 points out of a possible 100. A component score of less than 50 was defined as low, meaning that less than half the guideline recommendation was met. A high score was defined as 50 points or more. The cut-off value of 50 points out of a possible 100 was chosen after undertaking sensitivity analysis, using different values for the threshold. For example, other values considered included a score less than the average score for each component, a score less than 25% of the recommendation, and the lowest 10% and 30% of scores. Comparison of these results with the chosen threshold (50 out of 100) showed similar results, however the alternative approaches resulted in smaller cell counts. Using the data driven average value would have resulted in a different threshold for each component and population sample, making the communication in translation more complicated. All things considered, it was thought that a message of a low score being “less than half of the recommendation” was a relatively simple and clear message.

A value of one was assigned to a low score and a value of zero for a high score. Concatenating the values of ones and zeros of nine components in a standardised order created a nine-digit binary sequence that represented the dietary pattern of an individual (Table 1). In the example provided, the individual had three low scoring components in their dietary pattern with a low score for discretionary choices, vegetables and fruit. If this individual was a male in the 19–50 years age group, a score of less than 50 for fruit equated to consuming less than 1 serve of fruit per day, or for vegetables it equated to less than 3 serves of vegetables per day. For discretionary foods, the guideline recommendation is a maximum allowance, therefore a low score equated to consuming more than half the recommendation, which equated to more than 1.5 serves in this example.

To determine the number of low scoring components within a dietary pattern, the number of occurrences of the value one within the sequence were calculated. This number ranged from zero (no low scores within the sequence) to nine (all components had a low score). To examine the most common combinations of low scoring components in the sample, the frequency of nine-digit sequences was calculated. The most common dietary pattern sequences were thought to reflect the priority combinations of targets for intervention.

The frequency of nine-digit sequences were also calculated within subgroups of gender, age group, weight status and diet quality, and the five sequences with the highest occurrence within each subgroup are presented.

## 3. Results

### 3.1. Sample Characteristics

**Figure 1:**
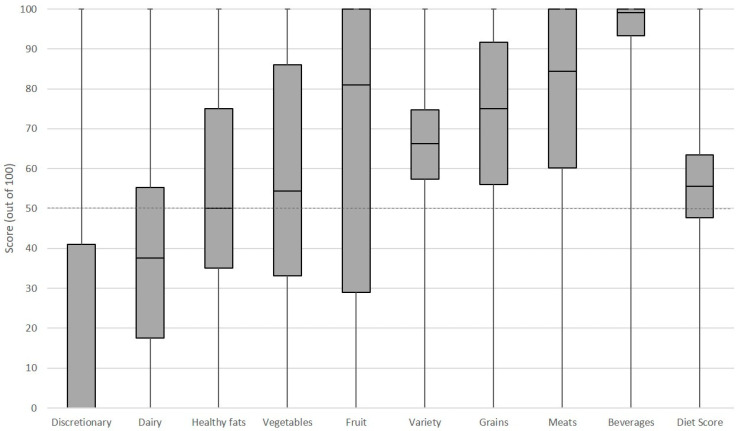
Box and whisker plot for the nine dietary guideline index components and total diet score (*n* = 230,575). The dotted line marks the cut-off for a low score of less than 50 points of a possible 100.

In this sample of 230,575 Australians, 51.2% were female, 35.1% and 29.5% aged between 31 to 50 years and 51 to 70 years, respectively. The calculation of body mass index showed about half the sample (45.4%) were of a healthy weight, while 32.7% were classified as overweight and 19.9% obese (Table 2). The mean and median diet score was 55.6 out of a possible 100, and about half the sample (50.3%) scored below this average. There was a large variation in scores with the observed values for all nine scoring components ranging from zero to 100 (Figure 1). The tendency for lower scores for discretionary choices, dairy, healthy fats, vegetables and fruit was evident from the 25th percentile value falling below 50 points out of 100, which was at least 20 points lower than the other components (Figure 1). In this sample, 79.5% of individuals had a low score for discretionary choices, 72.2% for healthy fats, 70.8% for dairy, 44.9% for vegetables and 41.4% for fruit (Table 2).

### 3.2. Overall Distribution of the Low Scoring Components

**Figure 2:**
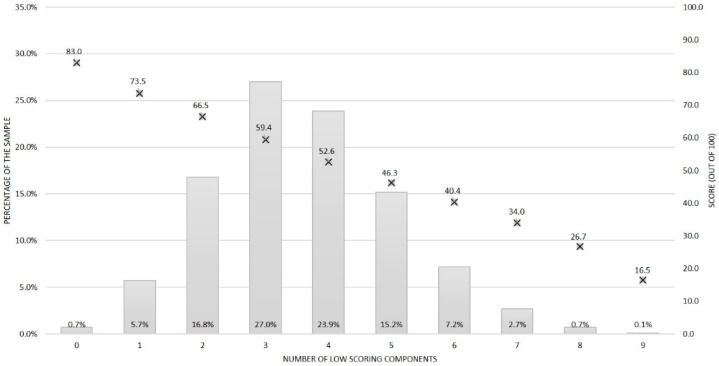
Distribution of the number of low scoring components within the dietary guideline index (bars) and total diet score (crosses) across the sample.

Figure 2 shows the distribution in the number of low scoring components within dietary patterns from the sample population. Most commonly, individuals had three or four low scoring components within their diet score (27.0% and 23.9% of the sample, respectively). Almost 83% of the sample had between two and five low scoring components within their diet score. Less than one percent of the sample had no (0.7%) or all (i.e., nine out of nine, 0.1%) low scoring components. The average diet score for individuals with five low scoring components was 46.3 compared to 66.5 for those with only two low scoring components. Males (29.7% of this subgroup), younger adults (33.3% of this subgroup) and individuals with obesity (30.7% of this subgroup) were more likely to have five or more low scoring components, but regardless of these demographic characteristics, having three or four low scoring components within the diet score was most common (Supplementary Table S1).

### 3.3. Combinations of Low Scoring Components

**Figure 3:**
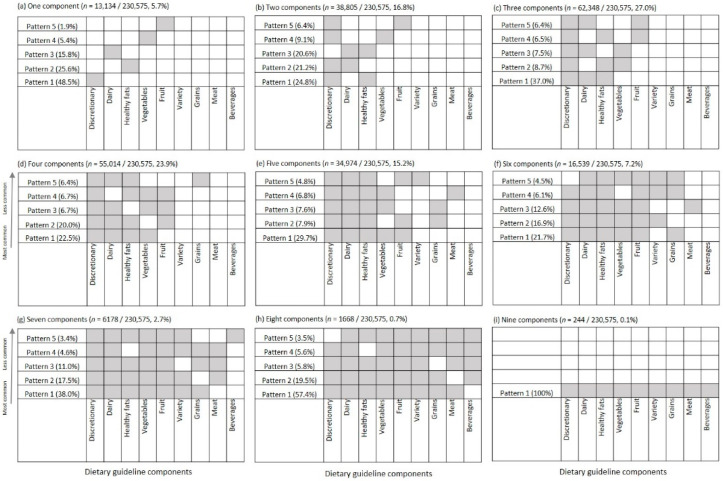
The five most common combinations of diet guideline index components, by total number of low scoring components. Data not shown for individuals with no low scoring components (*n* = 1670/230,575, 0.7% of the sample).

Figure 3 shows the most frequent combinations of diet quality components by the total number of low scoring components in the sample. For individuals with a total of two low scoring components within their dietary pattern (16.8% of the sample), these were mostly discretionary choices in combination with healthy fats (24.8% of this subsample), followed by discretionary choices with dairy (21.2%) or dairy with healthy fats (20.6%). Individuals with three low scoring components (27.0% of the total sample) most commonly had low scores on discretionary choices, dairy and healthy fats (37.0% of those with three low scoring components). The other combinations, although less common, were discretionary, healthy fats and vegetables (8.7%), or discretionary, dairy and vegetables (7.5%). The most common four-component combinations were discretionary, dairy, healthy fats and vegetables (22.5% of this subsample) or discretionary, dairy, healthy fats and fruit (20.0%). Individuals with a dietary pattern with five low scoring components had low scores for discretionary choices, dairy, healthy fats, fruit and vegetables (29.7% of this subsample, Figure 3). Amongst individuals with one to five low scoring components, 60.5% had a low score on one, all or a combination of discretionary, dairy, healthy fats, vegetables or fruit.

**Figure 4:**
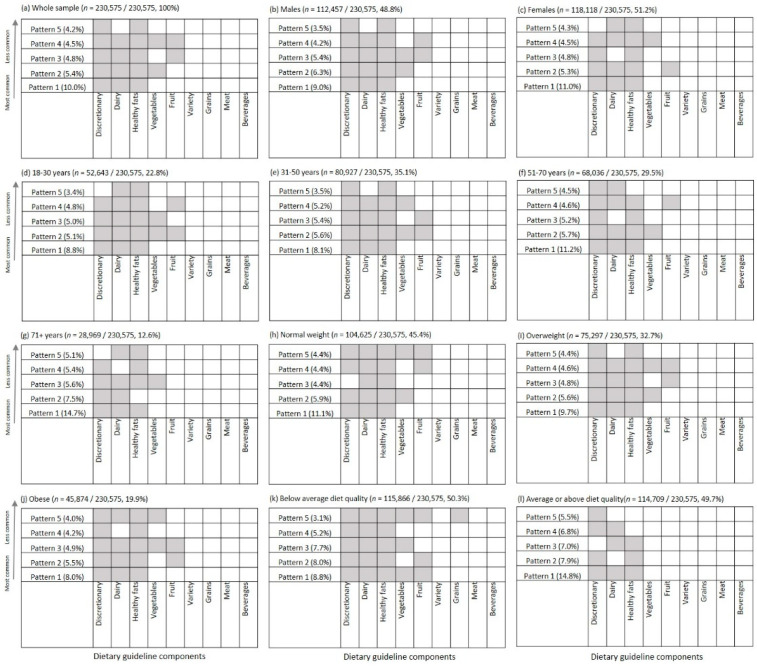
The five most common combinations of low scoring diet guideline index components, by gender, age group, weight status and diet quality. Data not shown for underweight individuals *n* = 4779/230,575, 2.1% of the sample).

Any specific combination of low scoring components was only observed in about 3 to 15% of any given subgroup (or approximately 11,000–34,000 out of 230,575 individuals), highlighting the variation in dietary patterns behind the dietary guideline index. However, discretionary, dairy, healthy fats, vegetables or fruit were the reoccurring low scoring components within most of the frequent combinations, regardless of the demographic subgroup of interest or the number of low scoring components. The most frequent combinations of low scoring components did not vary greatly by gender, age group or weight status (Figure 4). The three-component combination of discretionary choices, dairy and healthy fats was most common in all subgroups, except those with below average diet quality—where the five-component combination of discretionary, dairy, healthy fats, vegetables and fruit was most common (8.8% of the subgroup).

## 4. Discussion

This study aimed to describe the number and combination of low scoring components on a dietary guideline index within a large sample of Australian adults. Using composite index scores to indicate total diet quality of the population is important to understand how dietary patterns align with dietary guidelines. Examining the variation in total scores, as well as the specific components that make up a total score, can also deepen our understanding of population diet quality [25,26,27]. Within this sample, low scores on discretionary choices, healthy fats and dairy were common. The combinations approach showed that 83% of individuals had two to five low scoring components within their dietary guideline index score, with men, younger adults and individuals with obesity more likely to have five or more. This study was novel, as it examined low scores for single components as well as the combinations of low scoring components within a dietary guideline index score, to identify the common areas of poor eating habits in subgroups of the population. To the authors knowledge, no other studies have examined dietary guideline index scores in this way.

Around half of the sample had three or four low scoring diet quality components within their total score, and eight in ten people had between two and five low scoring components. Almost 80% of the sample had a low score for discretionary choices, and the discretionary choices appeared within the most common combinations more often than not. This was consistent with the Australian national level data demonstrating an excessive intake of discretionary choices, with the average reported intake being almost twice the maximum recommended intake [33]. Dairy foods was also in the commonly observed combinations, and national data suggest that 90% of adults are not meeting dairy recommendations [1]. Fruit and vegetables as a combination on its own was not among the most commonly observed, but they did feature with other food groups in combinations of four or more low scoring components. This was surprising, considering that national survey data suggest about 95% of adults fail to meet the dietary guidelines for vegetable intake, while more than three quarters fail to meet the guidelines for fruit intake [1]. The evidence for the benefits of fruit and vegetables for health and disease prevention is strong. They remain the cornerstone to a healthy diet and in turn are fundamental to dietary guidelines [23]. Given the importance of fruit and vegetables for health, public health interventions and campaigns tend to focus on increasing their consumption [3,13], however, the findings of this study suggest a need to broaden the targets for large-scale interventions to improve diet quality.

Targeting between two and five components of diet quality (out of nine) would address the poorest performing areas of most Australians’ diets and improve the overall compliance with the dietary guidelines. Discretionary choices, dairy and healthy fats was the most common low scoring combination (observed in 23,000 individuals or 10% of the sample). Vegetables and fruit, in addition to discretionary choices, dairy and healthy fats, were the low scoring components that made up the most common dietary pattern combinations observed. While there was considerable variation in the single component scores, there was consistency in the food groups that made up the most frequently observed combinations in this sample. The five food groups comprising the most common combinations of low scoring components (discretionary choices, dairy, healthy fats, vegetables and fruit) was consistent, regardless of an individual’s gender, age group, weight status or overall diet quality. These findings suggest that interventions that are flexible enough to address particular combinations of these food groups could be a useful approach to improving eating habits for individuals, and an effective approach to increase diet quality for a large proportion of the population. Changing dietary habits is complex, and as such interventions reported modest and mixed results. Some reviews reported positive changes in fruit and vegetable intake, and fat intake in various population groups [34,35,36]. However, changes in intake of other healthy food groups and discretionary foods and beverages is less consistent [34]. To achieve broader changes in dietary habits and develop more successful interventions, future research can learn from previous fruit and vegetable campaigns in terms of what worked well, such as a simple call to action message like “Go for 2 & 5” [16] or “five a day” [17,18,19], and determine how this can be applied to other food groups. The proposed food group targets from this study are diverse and different in how they are consumed in meals and snacks, therefore, campaigns to address their intake might require quite different approaches in large-scale campaigns.

There was consistency in the food groups that made up the common combinations, but there was variation in the total number of low scoring components within the dietary patterns of subgroups, therefore, specific population groups might require more attention. Findings suggest that men, younger adults and individuals with obesity were more likely to have five or more low scoring components, meaning they reached less than half the dietary guideline recommendation in most areas of diet quality assessed. Individuals with below average diet quality were also more likely to have low scores for more areas of their diet. Studies in the United States and Canada also found lower overall diet quality in younger adults and men, according to the Healthy Eating Index [37,38]. Interventions for these subgroups might need to target a greater range of dietary components in order to achieve substantial shifts in overall diet quality. Those designing interventions should consider that the difficulty and perceived complexity of adopting the suggested dietary changes might be greater for those whose current diet is furthest from the recommended pattern. As such an incremental approach to changing eating habits, where small changes are made one at a time, might be more appealing for these groups of people, easier to adopt and possibly more effective in improving the diet quality than broader whole-of-diet approaches [39].

A whole-of-diet approach has been positioned as the most important focus of healthy eating education [40], and can achieve significant health benefits for those who comply [41,42]. However, poor adherence to diets and retention on long-term lifestyle programs is a key challenge to the success of modifying dietary intake and other health behaviours [43,44]. In contrast, contemporary approaches to behaviour change encourage specification of target behaviours on the basis of need, rather than attempting to change too many behaviours at once [39]. This study identified specific combinations of food groups to target as priorities in interventions, to improve diet quality. A step-by-step approach has been utilised to good effect in small-scale interventions [15], and the findings of the present study could help to achieve similar results at a larger scale. Such an approach seems well suited to a digital platform that can be used to deliver a staged and flexible intervention [45]. While this study identified the targets, more research is required to understand the optimal journey through a dietary intervention of this nature. For example, does an individual’s diet quality improve more if they can choose the areas to address first, or can an automated approach achieve the desired change? It is likely going to be different for different people, and a digital platform would allow a more targeted nutrition intervention to be delivered at a scale that has not been previously undertaken.

### 4.1. Strengths and Limitations

Dietary guideline indices are useful to monitor population diet quality because they synthesise overall diet into one score. This is how indices are applied most often in research, however, the underlying components used to derive a total score can also be used as a tool to guide intervention design and develop targeted feedback. These applications of indices are not as well researched [26]. This analysis unpacked a dietary guideline index to understand the variation in the underlying dietary patterns in a large sample of 230,000 Australian adults, using data collected over the last 5 years. This sample size greatly exceeds that collected by other national nutrition surveys [9]. The analysis applied a novel approach by applying a cut-off to the dietary guideline index component scores to create binary variables of low/high scores and then used this to generate a nine-digit sequence representing an individual’s dietary pattern. Selecting less than 50 as the cut-off for a low score was thought to be a relatively simple and clear message, however, the choice of this value was somewhat arbitrary. Therefore, to understand the sensitivity of this choice as a threshold, additional analyses were conducted using different values as the threshold (such as less than the sample average for component scores). Comparison of these results with the current choice of threshold showed similar results in terms of the commonly occurring food group combinations, albeit with lower cell counts.

This analysis used data from a validated, online survey, where food intake was self-reported. There are limitations in surveys of this nature [28], however, to account for recruitment and self-report bias frequently observed with this kind of data collection, the reported food intake was adjusted and the sample data were weighted to reflect the gender and age distribution of the Australian population [31]. However, caution is needed with the interpretation of these findings in terms of their representativeness of the Australian population. The sample in this dataset differed on some characteristics compared to the broader Australian population, with a greater proportion of this sample being of a healthy weight (45% vs. 32% in the population), and as a result fewer in the obese category relative to the Australian population (20% vs. 31% in the population) [46]. This sample might be more health conscious, which could partially explain the higher consumption of fruit and vegetables relative to the national data. While the overall diet quality of this sample might be higher than the broader population, the most common observed dietary patterns reported here are likely similar to those within the population due to the consistency in the combinations observed across the different subgroups. It would have been ideal to make comparisons by socioeconomic status and other demographic characteristics such as ethnicity, however, such data are not currently collected through the CSIRO Healthy Diet Score survey.

### 4.2. Implications and Future Research

This study found that discretionary choices, healthy fats, dairy foods, fruit and vegetables are five areas of dietary guideline compliance that need improvement across all subgroups of the population examined. These results provide insights into ‘what’ food groups to target in order to improve diet quality, but further research into the most effective strategies around the ‘how’ to change dietary behaviour in this incremental way is needed. Small changes that are targeted, achievable and sustainable might be more effective, and possibly more resource efficient, in improving dietary intake compared to broad or complex dietary change [47]. For example, intervention strategies that start with a simple message like halve your snack food intake, or eat an extra piece of fruit each day, which then build to include messages from other food groups might be perceived as more achievable to individuals wanting to start changing their dietary habits. However, more understanding of ‘if’ and ‘how’ these small changes accumulate to improve overall diet quality is of interest. Small behavioural changes were shown to be an effective strategy for reducing energy intake, particularly when focusing on reductions in discretionary choices rather than increases in healthy foods and beverages [48]. However, substituting discretionary choices with healthy foods and beverages such as dairy, fruit, vegetables and wholegrains could have the dual effect of improving multiple low scoring dietary components simultaneously, potentially leading to more optimal impacts on nutrient adequacy, than moderation or reformulation of discretionary choices [49]. For example, strategies to replace sugar sweetened beverages with reduced fat-milk-based drinks could reduce discretionary beverage intake and increase compliance with the dietary guidelines for dairy foods by increasing the amount consumed as well as choosing “mostly reduced fat” dairy, which also forms part of the recommendation [5]; or replacing chocolate and confectionary for sweet tasting fruits could reduce discretionary food intake and increase fruit intake. Further research is needed to explore how these findings are translated into behaviour change strategies and effective interventions.

## 5. Conclusions

This study applied a unique analysis approach to identify common combinations of low scoring components within a dietary guideline index score, using data from a large sample of Australian adults. Discretionary choices, dairy, healthy fats, fruit and vegetables were the five areas of diet quality most in need of improvement, with most adults requiring intervention to address one or particular combinations of these food groups. Some subgroups of the population such as younger adults, men and people with obesity had more low scoring components within their dietary pattern. Findings suggest that focusing on these areas of diet quality would be beneficial for most of the population. Future research is required to determine the effectiveness of a targeted and flexible approach to improving overall diet quality, and how this more targeted food group approach can be optimised for large-scale delivery to achieve widespread changes in diet quality across the population.

## Figures and Tables

**Table 1:** Example of a nine-digit sequence of low (value = 1) and high (value = 0) component scores within an individual’s dietary guideline index score.

Position in Sequence	Diet Quality Component	Assigned Value
1	Discretionary choices	1
2	Dairy	0
3	Healthy fats	0
4	Vegetables	1
5	Fruit	1
6	Variety	0
7	Grains	0
8	Meat	0
9	Beverages	0
Dietary pattern sequence		10,011,000

**Table 2:** Demographics, weight status, diet quality characteristics and frequency of low scores for the dietary guideline index components in a sample of 230,575 Australian adults ^a^.

Characteristic	Categories	*n*	%
Total sample		230,575	100.0
Gender	Male	112,457	48.8
	Female	118,118	51.2
Age group	18–30 years	52,643	22.8
	31–50 years	80,927	35.1
	51–70 years	68,036	29.5
	71+ years	28,969	12.6
Weight status	Underweight	4779	2.1
	Healthy weight	104625	45.4
	Overweight	75,297	32.7
	Obesity	45,874	19.9
Diet quality score	Below average ^b^	115,866	50.3
	Average or above average	114,709	49.7
Low scoring components	Discretionary choices	183,220	79.5
	Healthy fats	166,364	72.2
	Dairy	163,171	70.8
	Vegetables	103,638	44.9
	Fruit	95,409	41.4
	Grains	46,244	20.1
	Meat	36,070	15.6
	Variety	29,514	12.8
	Beverages	7118	3.1

^a^ Data were weighted by gender and age group; ^b^ Below average diet quality was considered to be a score less than 55.6 out of a possible 100.

## Data Availability

The data presented in this study may be available on request from the corresponding author with restrictions. The data are not publicly available due to the nature of the consent provided by participants.

## Supplementary Materials

The following are available online at https://www.mdpi.com/1660-4601/18/2/378/s1. Table S1: Number of low scoring components within individuals’ dietary guideline index score by gender, age group, weight status and diet quality.
