# Supplementary material for: Understanding the Variation within a Dietary Guideline Index Score to Identify the Priority Food Group Targets for Improving Diet Quality across Population Subgroups
Source: Int J Environ Res Public Health. 2021 Jan 6;18(2):378. doi: 10.3390/ijerph18020378 (PMC7825319; doi:10.3390/ijerph18020378)
Supplement: Supplementary file 1 [file ijerph-18-00378-s001.pdf]

Table S1. Number of low soring components within individuals' dietary guideline index score by gender, age group, weight status and diet quality.

		0	1	2	3	4	5	6	7	8	9
Total sample	Total	0.7%	5.7%	16.8%	27.0%	23.9%	15.2%	7.2%	2.7%	0.7%	0.1%
Gender	Male	0.5%	4.7%	14.7%	25.5%	24.8%	17.1%	8.3%	3.3%	0.9%	0.1%
	Female	0.9%	6.7%	18.9%	28.5%	23.0%	13.3%	6.1%	2.1%	0.5%	0.1%
Age group	18–30 years	0.4%	3.8%	13.4%	24.4%	24.6%	17.8%	9.9%	4.1%	1.3%	0.2%
	31–50 years	0.4%	4.1%	13.4%	24.9%	25.7%	18.2%	9.0%	3.3%	0.9%	0.1%
	51-–0 years	1.0%	7.2%	20.2%	29.3%	22.9%	12.6%	4.8%	1.7%	0.3%	0.0%
	71+ years	1.5%	9.9%	24.6%	32.4%	19.6%	8.0%	2.8%	0.8%	0.2%	0.0%
Weight status	Underweight	0.6%	4.9%	15.3%	25.3%	23.4%	15.8%	8.0%	4.5%	1.8%	0.4%
	Normal weight	0.8%	5.9%	17.6%	28.1%	23.7%	14.4%	6.5%	2.3%	0.6%	0.1%
	Overweight	0.7%	5.8%	16.8%	27.1%	24.3%	15.1%	6.9%	2.5%	0.6%	0.1%
	Obese	0.6%	5.2%	15.2%	24.8%	23.5%	16.8%	9.0%	3.6%	1.1%	0.2%
Diet quality	Below average	0.0%	0.0%	1.5%	15.4%	33.7%	28.2%	14.2%	5.3%	1.4%	0.2%
	Average or above average	1.5%	11.4%	32.3%	38.8%	13.9%	2.0%	0.1%	0.0%	0.0%	0.0%
